# Self-management of incontinence using a free mobile app: factors associated with improvement

**DOI:** 10.1007/s00192-021-04755-5

**Published:** 2021-04-07

**Authors:** Emma Nyström, Lars Söderström, Eva Samuelsson

**Affiliations:** 1 Department of Public Health and Clinical Medicine, Umeå University, The Research Unit, Region Jämtland Härjedalen, Östersund, SE-831 27 Sweden; 2 Unit of Research, Education and Development, Östersund, Sweden

**Keywords:** Mobile applications, Pelvic floor muscle training, Predictors, Self-management, Urinary incontinence

## Abstract

**Background:**

Pelvic floor muscle training (PFMT) is first-line treatment for urinary incontinence (UI) in women. Self-management via a mobile app is a new cost-effective method for PFMT delivery. This study analyzes factors associated with improvement among app users.

**Methods:**

A pragmatic observational study in a community setting. Upon downloading the app Tät®, users answered questions regarding their age, education, residence, and UI symptoms. After 3 months, users answered follow-up questions regarding symptoms and frequency of training and app usage, and the validated Patient Global Impression of Improvement (PGI-I) questionnaire. Only non-pregnant, non-postpartum adult women with UI who answered the PGI-I questionnaire were included. Multivariate logistic regression was used to analyze possible associations between these factors with any improvement and with great improvement according to the PGI-I. The models were adjusted for age.

**Results:**

The study included 2,153 participants who had completed self-management, that is, 11.5% of eligible women who completed the baseline questionnaire. Of these participants, 65.6% reported improvement of UI. Any improvement was associated with age, frequency of PFMT, and app use, accounting for 27.9% of variability (Nagelkerke R^2^). Lower incontinence severity, frequency of PFMT, and app use were associated with great improvement.

**Conclusion:**

Self-management of urinary incontinence is easily accessible to many women and improvement rates are comparable with other forms of PFMT. Demographic factors and incontinence severity showed no or incongruent association, whereas regular PFMT and app use predicted any and great improvement. App use showed an additional effect beyond frequency of training.

## Introduction

Urinary incontinence is common among women, with reported prevalence rates most frequently ranging from 25 to 45% [1]. For all types of urinary incontinence among women, the first-line treatment includes pelvic floor muscle training (PFMT) and lifestyle advice [2]. The most recent Cochrane review concludes that 67% of women with any type of urinary incontinence who perform PFMT report cure or improvement, which is twice as many as women in control groups [3]. Furthermore, PFMT is six times as likely to lead to cure or improvement in women with stress urinary incontinence (SUI), i.e., the most prevalent type of incontinence, compared with controls [3].

Pelvic floor muscle training can be provided through supervised or self-directed programs, and it is recommended that the most intensive program possible be offered [2]. One review suggests the initiation of unsupervised PFMT and recommends using a modality that facilitates compliance [4]. One method of providing unsupervised PFMT safely and effectively is via eHealth, such as a mobile app [5]. The app Tät® has been found to have short- and long-term effects on symptoms and quality of life in a randomized controlled trial (RCT) [6, 7], and is also a cost-effective means of management [8]. It has also received high ratings in recent comparisons of mobile apps [9].

Some studies imply that background factors, such as age [10, 11] and educational level [12, 13], can influence the outcome following various types of behavioral training, including PFMT. However, these studies have not confirmed each other’s results, and the identified associations depend on the definition of treatment success [12, 13]. Other larger studies show no association between treatment success and age or education, but report that less severe leakage at baseline predicts success [14, 15]. Treatment adherence is considered crucial for treatment effect, but further studies are needed to investigate the association between adherence and treatment outcome [16]. Only small studies have examined the factors associated with success when using PFMT-focused eHealth interventions, such as mobile-based or Internet-based management [10, 17]. One of these studies examined the Tät® app, but was too small to offer any guidance on whether some women are more likely to benefit than others.

Overall, there remains a need for studies to determine who benefits of PFMT in general, what level of adherence is critical, and who benefits specifically from freely available mobile-based self-management.

In the present study, we aimed to determine whether background factors and adherence were associated with improvement of urinary incontinence from self-management via a mobile app focused on PFMT.

## Materials and methods

This investigation was part of a pragmatic observational study of the effects of mobile-based self-management for urinary incontinence. The app Tät® was designed for self-management of stress urinary incontinence in women. It includes information, lifestyle advice, PFMT exercises with graphical support, reminders, and a statistical function. The graphical support consisted of a moving bar, indicating the contraction time and intensity the user should aim for. The efficacy of Tät® was demonstrated in an RCT [6], after which the app was made freely available in Swedish from the App store or Google Play. Subsequently, authorized translators have translated the app into English, Finnish, German, Spanish, and Arabic, and the app has been released in those languages as well. The overall aim of the pragmatic study was to describe the use and effectiveness of the app when it was used under real-life circumstances.

Upon downloading the app, the user was informed about the study of the app when freely available, and was invited to participate by answering a short baseline questionnaire. Participants who were still using the app after 3 months were asked to complete a follow-up questionnaire. The study information and the questionnaires were also translated by authorized translators and appeared in the language that had been chosen when users downloaded the app. Both questionnaires were anonymously submitted to a secure research database. The questionnaires were assigned a unique identification number generated within the app, such that they could be correctly paired but not traced back to a specific mobile phone, telephone number, or any other personal data. Hence, all study data were self-reported, and we had no ability to confirm any of the submitted information.

Data were collected from participants who had downloaded the app in all available languages between 16 January 2018 and 1 June 2019. Another article describes this population, as well as the characteristics of completing participants and their effect on incontinence symptoms [18].

**Fig. 1:**
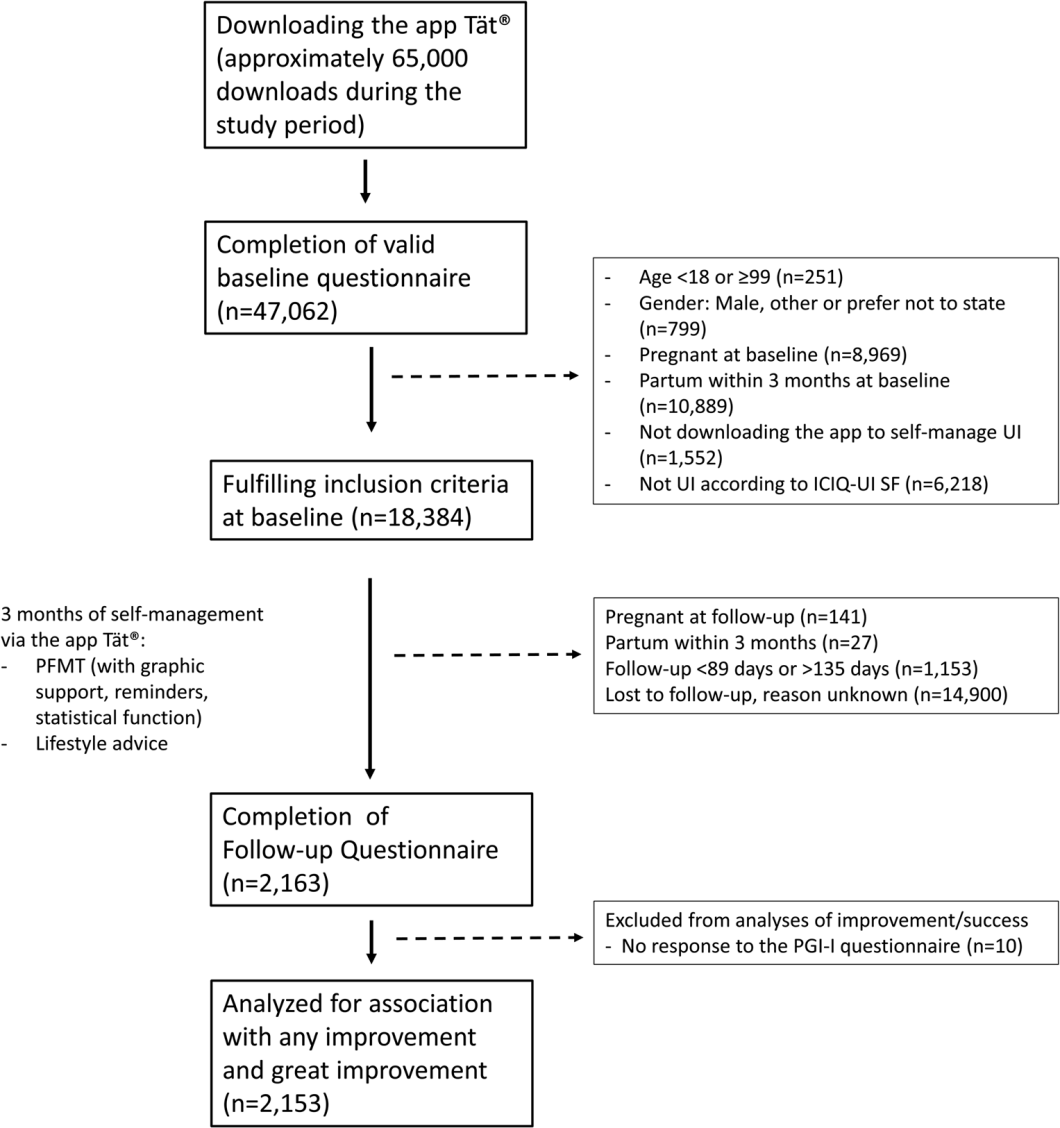
Flow diagram of the study

In this present study we only included those participants who had answered the PGI-I within 89–135 days (Fig. 1). Questionnaires submitted after more than 135 days were considered to reflect a more sporadic opening of the app rather than the continuous use that we aimed to study, or a reluctance to participate in the study and hence postponing the response. Follow-up time of shorter than 89 days was considered to indicate a technical error as this should not have been possible.

Further inclusion criteria were self-stated female sex, age of ≥18 years, and urinary incontinence defined according to the validated International Consultation on Incontinence Questionnaire-Urinary Incontinence Short Form (ICIQ-UI SF). Participants were considered to have urinary incontinence if they reported both leakage on the ICIQ-UI SF question “How often do you leak urine?” and if they reported any amount to the ICIQ-UI SF question “How much urine do you usually leak?” Exclusion criteria were current pregnancy or post-partum (<3 months) at baseline or follow-up.

### Ethical approval and informed consent

The Regional Ethical Review Board, Umeå University, granted ethical approval to both collect the data (number 2014–389-32 M) and perform specific analyses in this study (number 2017–405-32 M). After reading the provided information about the study, it was optional to complete the questionnaire. Submission of the questionnaire was considered to indicate consent to participate, and these users were included in the study. No reimbursement was given.

### Questionnaires at baseline and 3-month follow-up

Upon first opening the app, participants answered a baseline questionnaire about age, educational level, and current residence, as well as the validated questionnaire assessing urinary incontinence symptoms (ICIQ-UI SF) [19]. Age was registered as the stated age at baseline, and then divided into four categories: <30 years, 30–39 years, 40–49 years, and ≥ 50 years. To report their current residence, users answered a multiple-choice question with answers ranging from metropolitan to rural area, and could state their country of residence. The variable education was then recoded into a dichotomous variable: university education or not, because there were very few participants who had had 9 years or less of education. Questions were also asked about current pregnancy or recent delivery (<3 months) at baseline and follow-up. Three months after answering the baseline questionnaire, users were asked to complete the symptom score ICIQ-UI SF again, to estimate their frequency of training over the last 4 weeks and app usage since they downloaded the app, and to rate their improvement using the validated questionnaire Patient Global Impression of Improvement (PGI-I) [20]. The questions about PFMT frequency and app use had five different answer categories, as reported in Tables 1, 2, 3, which were not altered in any way for the analyses.

**Table 1:** Baseline and follow-up characteristics analyzed for association with any and great improvement (*N* = 2,153)

Factors	All participants
Baseline factors	
Age, *n* (%)	
<30 years	171 (8.0)
30–39 years	672 (31.2)
40–49 years	567 (26.3)
≥50 years	743 (34.5)
Education, *n* (%)	
≤ 9 years of school	57 (2.6)
10–12 years of school	462 (21.5)
University or college	1,634 (75.9)
Residence, *n* (%)	
Rural area	364 (16.9)
Urban area < 50,000 inhabitants	568 (26.4)
Urban area 50,000–1 million inhabitants	837 (38.9)
Metropolitan area ≥ 1 million inhabitants	384 (17.8)
Type of incontinence, *n* (%)	
Stress urinary incontinence	1,192 (55.4)
Mixed urinary incontinence	630 (29.3)
Urgency urinary incontinence	261 (12.1)
Other incontinence	70 (3.2)
ICIQ-UI SF, mean (SD)	8.13 (3.87)
Factors at follow-up	
Frequency of PFMT, *n* (%)	
No, never	189 (8.8)
Less than once a week	509 (23.6)
1–6 times a week	818 (38.0)
Every day	516 (24.0)
Three times a day or more	121 (5.6)
Usage of the app, *n* (%)	
Have not used it at all	234 (10.9)
About once a month	303 (14.1)
About once a week	480 (22.3)
About once a day	602 (27.9)
Several times a day	534 (24.8)

*ICIQ-UI SF* International Consultation on Incontinence Modular Questionnaire-Urinary Incontinence Short Form, *PFMT* pelvic floor muscle training

**Table 2:** Univariate analysis of baseline and follow-up factors (*N* = 2,153)

Factors		Association with any improvement		Association with great improvement	
		p*	Crude OR (CI)*	p*	Crude OR (CI)*
Baseline factors					
Age					
<30 years		Reference	1.0	Reference	1.0
30–39 years		0.282	0.83 (0.58–1.17)	0.479	0.87 (0.59–1.29)
40–49 years		0.799	0.95 (0.67–1.37)	0.658	0.91 (0.61–1.36)
≥50 years		0.073	1.38 (0.97–1.96)	0.042	1.48 (1.01–2.17)
Education					
12th grade or lower		Reference	1.0	Reference	1.0
University or college		0.624	1.05 (0.86–1.30)	0.388	0.91 (0.73–1.13)
Dwelling					
Rural area		Reference	1.0	Reference	1.0
Urban area <50,000 inhabitants		0.714	0.95 (0.72–1.25)	0.915	1.02 (0.76–1.36)
Urban area 50,000 to 1 million inhabitants		0.764	0.96 (0.74–1.25)	0.142	0.81 (0.61–1.07)
Metropolitan area ≥1 million inhabitants		0.747	0.95 (0.70–1.29)	0.440	0.88 (0.64–1.22)
Type of incontinence					
Stress urinary incontinence		Reference	1.0	Reference	1.0
Mixed urinary incontinence		0.648	1.07 (0.80–1.42)	0.024	1.40 (1.04–1.88)
Urgency urinary incontinence		0.572	1.06 (0.86–1.30)	0.522	1.07 (0.86–1.34)
Other incontinence		0.522	0.85 (0.52–1.40)	0.206	1.40 (0.83–2.36)
ICIQ-UI SF		0.783	1.00 (0.98–1.03)	0.014	0.97 (0.94–0.99)
Factors at follow-up					
Frequency of PFMT					
No, never		Reference	1.0	Reference	1.0
Less than once a week		<0.001	2.24 (1.57–3.19)	0.494	1.19 (0.73–1.95)
1–6 times a week		<0.001	5.45 (3.86–7.69)	0.001	2.24 (1.42–3.54)
Every day		<0.001	10.53 (7.18–15.45)	<0.001	4.35 (2.74–6.92)
Three times a day or more		<0.001	21.04 (10.74–41.20)	<0.001	7.22 (4.14–12.61)
Usage of the app					
Have not used it at all		Reference	1.0	Reference	1.0
About once a month		0.002	1.82 (1.25–2.66)	0.379	0.80 (0.48–1.32)
About once a week		<0.001	5.36 (3.77–7.62)	0.594	1.13 (0.72–1.76)
About once a day		<0.001	13.01 (9.07–18.66)	<0.001	2.81 (1.87–4.22)
Several times a day		<0.001	17.62 (12.03–25.81)	<0.001	4.57 (3.05–6.86)

*CI* confidence interval, *ICIQ-UI SF* International Consultation on Incontinence Modular Questionnaire-Urinary Incontinence Short Form, *OR* odds ratio, *PFMT* pelvic floor muscle training

*Results from univariate logistic regression

**Table 3:** Factors associated with any and great improvement (*N* = 2,153)

Significant factors in the multivariate model	Association with any improvement		Association with great improvement	
	p*	Adjusted OR (CI)*	p*	Adjusted OR (CI)*
Age				
<30 years	Reference	1.0	Reference	1.0
30–39 years	0.002	0.54 (0.36–0.80)	0.056	0.67 (0.44–1.01)
40–49 years	0.015	0.60 (0.40–0.91)	0.090	0.69 (0.45–1.06)
≥50 years	0.141	0.74 (0.49–1.11)	0.912	0.98 (0.65–1.47)
Incontinence severity				
Total ICIQ-UI SF score		NS	<0.001	0.95 (0.92–0.97)
Pelvic floor muscle training				
Never	Reference	1.0	Reference	1.0
Less frequent than once per week	0.008	1.72 (1.15–2.56)	0.422	1.24 (0.74–2.08)
1–6 times a week	<0.001	2.33 (1.56–3.47)	0.056	1.63 (0.99–2.71)
Daily	<0.001	2.88 (1.83–4.54)	0.006	2.10 (1.24–3.55)
Several times a day	<0.001	5.41 (2.57–11.37)	<0.001	3.13 (1.68–5.85)
Usage of app				
Never used the app	Reference	1.0	Reference	1.0
About once a month	0.009	1.68 (1.14–2.50)	0.337	0.77 (0.46–1.31)
About once a week	<0.001	4.38 (3.01–6.37)	0.916	0.97 (0.61–1.57)
About once a day	<0.001	9.20 (6.22–13.60)	0.001	2.10 (1.34–3.29)
Several times a day	<0.001	10.35 (6.73–15.90)	<0.001	2.93 (1.84–4.67)

*CI* confidence interval, *ICIQ-UI SF* International Consultation on Incontinence Modular Questionnaire-Urinary Incontinence Short Form, *OR* odds ratio, *PFMT* pelvic floor muscle training

*Results from the multivariate logistic regression model adjusted for age. *p* < 0.05 was considered significant

The questions were considered as factors potentially associated with treatment outcome and were thus analyzed for possible association. Age was analyzed as a continuous and categorical variable in the univariate analyses. Education level, residence, and frequency of PFMT and app usage were analyzed as categorical variables. The total ICIQ-UI SF score was analyzed as a continuous variable.

### Treatment outcome

We analyzed the selected factors to determine their impact on two different levels of treatment outcome: any improvement after 3 months and great improvement after 3 months. These were defined as follows:

1. Any improvement at follow-up was defined as giving an answer to the PGI-I questionnaire that indicates improvement that is “a little better,” “much better,” or “very much better.”
2. Great improvement at follow-up was defined as rating one’s condition as “much better” or “very much better” according to the PGI-I questionnaire.

These outcomes were chosen for two reasons: first, to ensure a patient-centered approach and second, the definition of great improvement has been used in other studies [10, 13, 17]. Owing to the large sample size, we chose to analyze the impact of the factors on two levels of improvement to better describe the impact of the factors and thus avoid drawing conclusions from randomly significant factors.

### Statistical analysis

The factors were first assessed using descriptive analysis to summarize and describe demographics and baseline characteristics. Data were further analyzed using the Pearson Chi^2^ or Fisher’s exact test, as appropriate, and univariate logistic regression to assess possible associations with outcome. Next, regardless of univariate associations, the factors were all included in a multivariate logistic regression model. We analyzed separate multivariate logistic regression models for each outcome variable. For both outcomes, the following factors were entered into the multivariate regression model: age, educational level, current residence, total ICIQ-UI SF score, type of incontinence, frequency of PFMT, and frequency of app use. Factors were stepwise removed according to significance level until only significant variables remained (*p* ≤ .05). Age was included as a categorical variable in the multivariate analyses, as it better described the relationship with the outcome. Throughout the analysis, age was included in the models to control for possible age differences.

As the app is used to support PFMT, the variables “frequency of PFMT” and “frequency of app use” were analyzed for correlation and the models were also analyzed with these variables separately to find which variable better describes the relationship with the different levels of improvement.

As the questionnaires could only be submitted if all the questions were answered, there was no missing data from the completing participants. All statistical analyses were performed using SPSS version 26 software.

## Results

During the study period, a total of 2,153 women with urinary incontinence submitted the baseline and follow-up questionnaire, including a response to the PGI-I question via the app Tät®. These women were included in this study and amounted to 11.5% of all eligible women who had downloaded the app for self-management and completed the baseline questionnaire (Fig. 1).

Of these participants, 1,973 used the Swedish version of the app, 111 the English version, and 69 had used the app in other languages. App users reported 34 different countries of residence, including Sweden. The average user age was 45.6 years (SD 13.3 years), and more than three-quarters were studying or had completed college or university education. The vast majority (84.6%) had symptoms of stress or mixed urinary incontinence (Table 1).

The mean follow-up time was 101.3 days (SD 12.5 days). The vast majority of users (67.6%) had performed pelvic floor muscle training at least weekly during the last 4 weeks, and 75.1% had used the app at least weekly throughout the self-management period. Table 1 presents additional baseline and follow-up data, and Table 2 describes their unadjusted association with the outcomes.

### Factors associated with any improvement

**Fig. 2:**
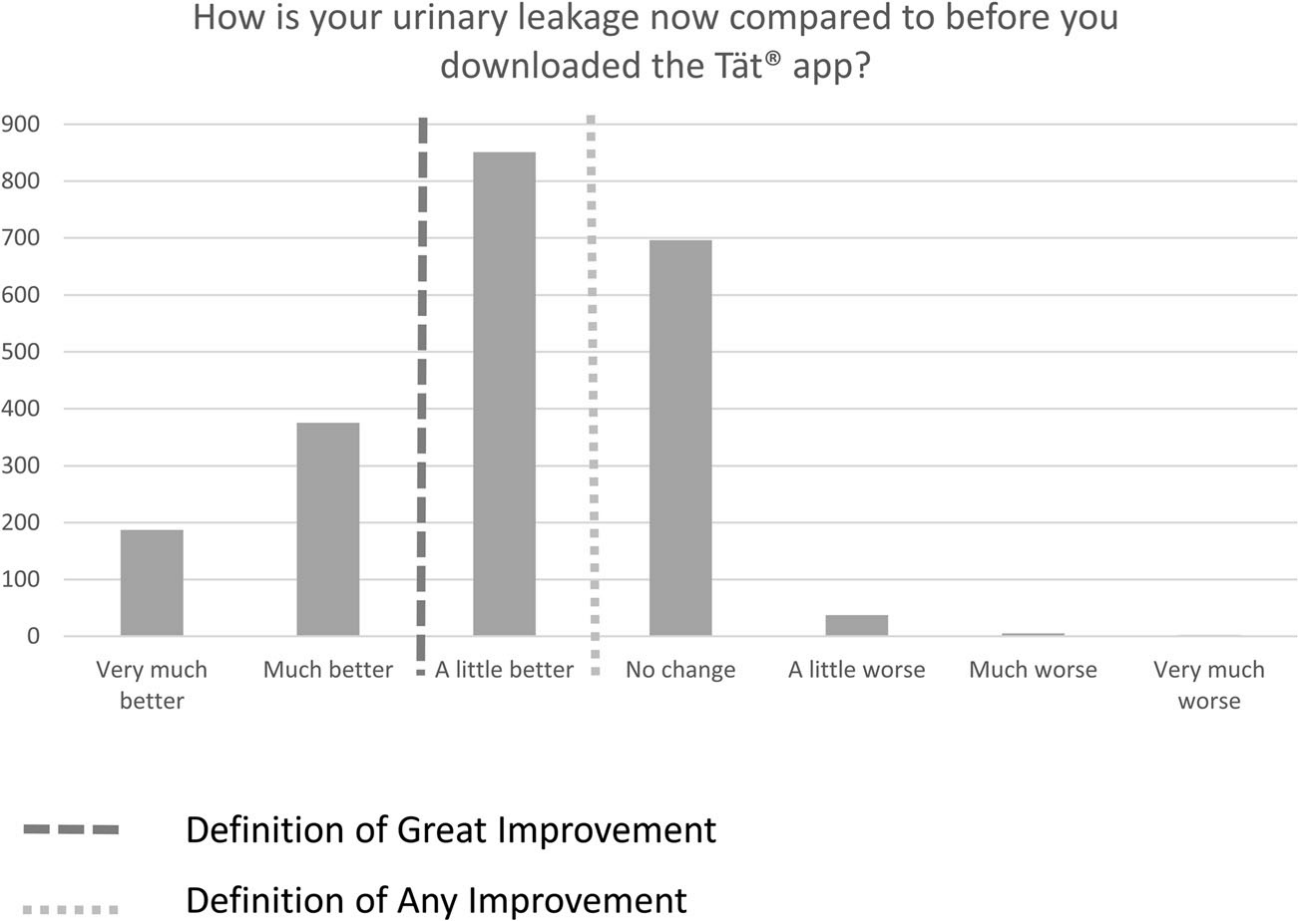
Patient Global Impression of Improvement (PGI-I) at follow-up after 3 months of self-management and definition of the outcome variables

At follow-up, 1,413 participants (65.6%) rated their condition to be a little better, much better, or very much better, and were thus considered to exhibit any improvement (Fig. 2). For the outcome “any improvement”, the final model revealed significant associations with the following variables: age, PFMT frequency, and app usage frequency (Table 3). Together, these factors explained 27.9% of the variability in improvement (Nagelkerke R^2^). The association with age was not linear; both younger and older age increased the odds. Higher odds of perceiving any improvement were associated with more frequent PFMT and app usage, but there was no significant difference between weekly and daily PFMT or app use (Table 3). Furthermore, less frequent but regular PFMT and app use significantly increased the odds compared with no training/use of the app.

### Factors associated with great improvement

Among participants who improved, 562 (26.1% of all who completed the PGI-I) perceived their condition to be much or very much better, and were categorized as having a great improvement (Fig. 2). The outcome “great improvement” was found to have a significant association with only three variables: total ICIQ-UI SF score, PFMT frequency, and app usage frequency (Table 3). Together, these factors explained 14.0% of the variability (Nagelkerke R^2^). More frequent PFMT and app use increased the odds of great improvement; however, the confidence intervals of the categories of regular training/use overlapped and only daily training and app use increased the odds significantly from no training/no use (Table 3).

### Correlation of PFMT frequency and app usage

We identified a substantial correlation between PFMT frequency and app usage (Spearman rho = 0.633, *p* < 0.001). When only one of these factors was included in the model, the variable app usage predicted the outcome better, but not as well as the combination. As it was likely that the impact of app usage was partly a reflection of the impact of PFMT frequency, we included PFMT frequency in both models to control for the impact of PFMT alone.

## Discussion

In the present study, we found that among the 2,153 participants who completed 3 months of self-management, 65.6% felt that their condition had improved. Age was associated with any improvement at 3 months, but not with great improvement. Incontinence severity (total ICIQ-UI SF score) was only associated with great improvement. Finally, PFMT frequency and app usage were associated with both levels of improvement.

### Strengths and limitations

Strengths of this study include the evidence-based intervention, the large sample size, and the prospective study design. Furthermore, this study investigated a new method of delivering a training program for urinary incontinence, and a first step in incontinence care that has the potential to reach a broad population. Moreover, this study was conducted in the exact clinical setting in which the self-management method is intended to be used.

The pragmatic study design was also the most important limitation. To make it easy for users to participate, the questionnaires were submitted electronically, and to ensure data security, they could not be traced back to the participant. Therefore, we had no means of validating the data or of reminding participants who discontinued. With all self-reported data there is always a risk of recall bias; we tried to minimize this by not allowing follow-up times that were too lengthy and by allowing women to see their actual PFMT frequency in the statistics function of the app. The short questionnaire also meant that there were several factors that could not be controlled for, such as parity and previous incontinence surgery.

### Discontinuation and adherence

A minority (11.5%) of the eligible women who completed the baseline questionnaire responded to the follow-up questionnaire. It has been described that women self-manage with daily PFMT for a long time before seeking help [21]. Some participants may have continued to perform PFMT without the support of the app after reading the initial instructions. Others may not have been prepared to invest the time and effort required to actively self manage. Women who perceive their symptoms to be less bothersome are less likely to seek care [22]. As eHealth lowers the barrier to seeking help [23], our population probably also included women who perceived their symptoms to be less bothersome and were less motivated to perform PFMT. Another study of this current population showed that women with more advanced age and higher level of education, as well as Swedish and incontinent users, completed follow-up to a greater extent, but that the severity and the type of incontinence of the incontinent users had no impact on follow-up rates [18].

The completion and improvement rates in this study were lower than in the RCT of the same app [6]. In that population, 98% of users completed follow-up and 92% of them reported any improvement and 56% great improvement [6]. However, the participants in the RCT had to go through an extensive enrolment process and it is likely that the participants that passed this process (15%) were more motivated to exercise than our study population. In the RCT, 41.0% of participants had performed PFMT daily [6], compared with 29.6% in our population. In qualitative analysis of the app users’ experience the reminder within the app was found to be helpful [24]. As many participants never completed the follow-up, there could also be a need for reminders or follow-up outside of the app.

### Our findings in relation to previous data

Age was associated with any improvement, but this relationship was not linear. The association with younger age is in line with previous findings in women receiving drug treatment or combined treatment for UUI [25]. The association with older age is in line with what Lindh et al. found for PFMT via the Internet [10] and with the findings of Labrie et al., with higher rates of crossover to surgery among younger women [26]. The incongruence in these findings is probably due to the different outcomes used, which also depend very much on the expectations of the women, and the non-linear relationship we found between age and improvement.

A higher ICIQ-UI SF score decreased the odds of great improvement, which has also been found by other studies [10, 12, 14, 27]. However, this also depends on the outcome used, for example, Lindh et al. found that women with higher ICIQ-UI SF scores were more likely to have ≥3 points reduction but less likely to be satisfied with treatment [10]. In line with this, Burgio et al. report that women with higher incontinence episode frequency are less likely to be completely dry after behavioral training [12], and Labrie et al. report that higher incontinence severity predicts crossover to surgery [26].

In this present study a higher educational level was not associated with any level of improvement. In another study of this cohort it was associated with higher rates of self-management completion [18], but that finding may reflect the overall higher use of health apps among highly educated individuals [28]. Investigations of educational level as a predictor of treatment success among participants with urinary incontinence have found divergent results. In women with symptoms of stress urinary incontinence, higher educational level has been found to be associated with treatment success according to the Urogenital Distress Inventory but not according to the PGI-I [13]. In contrast, in a cohort with more urgency urinary incontinence lower educational level has been found to predict total continence [12]. These prior findings and our present results suggest that educational level might have different impacts on different outcomes and in different subpopulations, but that the overall effect is likely small.

Our findings regarding higher PFMT adherence and use of the app further support previous suggestions that treatment adherence is essential for improvement [16]. With another form of self-management, internet-based PFMT, at least weekly PFMT was a predictor of long-term success [10]. The additional value of app use found in our study may be attributable to the information and lifestyle advice that is also part of the recommended treatment [2, 4]. Current recommendations indicate that the most intense training should be offered [2]. Training with the app may have been more intense, as it enables the user to reread the instructions, and provides graphic support that may improve training quality. This, along with other factors we could not control for, could also explain part of the overlap between the weekly and daily training. There could also be other reasons, for example, it is likely that weekly training of good quality is better than daily training of poor quality, and that the frequency of training alone is an insufficient measure of adherence.

### Clinical implications

These results show that a free mobile app can reach many women with urinary incontinence and that improvement rates are comparable with other forms of PFMT [3] if 3 months of active self-management is completed. The results do not indicate that app management should only be offered to certain groups, and they support the previously established importance of adherence. However, although self-management via a mobile app is a potentially cost-effective [8] first-line treatment for many women, others may need more support or other forms of treatment to complete 3 months of PFMT.

### Future directions

As self-management or other unsupervised PFMT is the most feasible way of providing a prompt treatment option to many patients, additional studies should evaluate how best to improve adherence and training quality with unsupervised training programs.

## Conclusions

Self-management of urinary incontinence via a mobile app is an effective means of first-line management, which can easily reach a large number of people. Demographic factors and incontinence severity offer no support for only recommending self-management to certain groups. Our results indicate that regular pelvic floor muscle training and use of the app during self-management are the most important factors for achieving improvement. The app also includes lifestyle advice, reminders, graphical support, and other functions, which may explain the additional benefits of app use.

## References

[1] Milsom I, Altman D, Cartwright R, Lapitan MC, Nelson R, Sjöström S, Abrams P, Cardozo L, Wagg A, Wein A (2017). Epidemiology of urinary incontinence (UI) and other lower urinary tract symptoms (LUTS), pelvic organ prolapse (POP), and anal incontinence (AI). Incontinence.

[2] Dumoulin C, Adewuyi T, Booth J, Bradley C, Burgio K, Hagen S, Abrams P, Cardozo L, Wagg A, Wein A (2017). Adult conservative management. Incontinence.

[3] Dumoulin C, Cacciari LP, Hay-Smith EJC (2018). Pelvic floor muscle training versus no treatment, or inactive control treatments, for urinary incontinence in women. Cochrane Database Syst Rev.

[4] Lukacz ES, Santiago-Lastra Y, Albo ME, Brubaker L (2017). Urinary incontinence in women: a review. JAMA.

[5] Novara G, Checcucci E, Crestani A, Abrate A, Esperto F, Pavan N (2020). Telehealth in urology: a systematic review of the literature. How much can telemedicine be useful during and after the COVID-19 pandemic?. Eur Urol.

[6] Asklund I, Nyström E, Sjöström M, Umefjord G, Stenlund H, Samuelsson E (2017). Mobile app for treatment of stress urinary incontinence: a randomized controlled trial. Neurourol Urodyn.

[7] Hoffman V, Söderström L, Samuelsson E (2017). Self-management of stress urinary incontinence via a mobile app: two-year follow-up of a randomized controlled trial. Acta Obstet Gynecol Scand.

[8] Sjöström M, Lindholm L, Samuelsson E (2017). Mobile app for treatment of stress urinary incontinence: a cost-effectiveness analysis. J Med Internet Res.

[9] Barnes KL, Dunivan G, Jaramillo-Huff A, Krantz T, Thompson J, Jeppson P (2019). Evaluation of smartphone pelvic floor exercise applications using standardized scoring system. Female Pelvic Med Reconstr Surg.

[10] Lindh A, Sjöström M, Stenlund H, Samuelsson E (2016). Non-face-to-face treatment of stress urinary incontinence: predictors of success after 1 year. Int Urogynecol J.

[11] Weinberger MW, Goodman BM, Carnes M (1999). Long-term efficacy of nonsurgical urinary incontinence treatment in elderly women. J Gerontol A Biol Sci Med Sci.

[12] Burgio KL, Goode PS, Locher JL, Richter HE, Roth DL, Wright KC (2003). Predictors of outcome in the behavioral treatment of urinary incontinence in women. Obstet Gynecol.

[13] Schaffer J, Nager CW, Xiang F, Borello-France D, Bradley CS, Wu JM (2012). Predictors of success and satisfaction of nonsurgical therapy for stress urinary incontinence. Obstet Gynecol.

[14] Hendriks EJ, Kessels AG, de Vet HC, Bernards AT, de Bie RA (2010). Prognostic indicators of poor short-term outcome of physiotherapy intervention in women with stress urinary incontinence. Neurourol Urodyn.

[15] Cammu H, Van Nylen M, Blockeel C, Kaufman L, Amy JJ (2004). Who will benefit from pelvic floor muscle training for stress urinary incontinence?. Am J Obstet Gynecol.

[16] Dumoulin C, Hay-Smith J, Frawley H, McClurg D, Alewijnse D, Bo K (2015). 2014 consensus statement on improving pelvic floor muscle training adherence: International Continence Society 2011 state-of-the-science seminar. Neurourol Urodyn.

[17] Nyström E, Asklund I, Sjöström M, Stenlund H, Samuelsson E (2018). Treatment of stress urinary incontinence with a mobile app: factors associated with success. Int Urogynecol J.

[18] Rygh P, Asklund I, Samuelsson E (2021). Real-world effectiveness of app-based treatment for urinary incontinence: a cohort study. BMJ Open.

[19] Avery K, Donovan J, Peters TJ, Shaw C, Gotoh M, Abrams P (2004). ICIQ: a brief and robust measure for evaluating the symptoms and impact of urinary incontinence. Neurourol Urodyn.

[20] Yalcin I, Bump RC (2003). Validation of two global impression questionnaires for incontinence. Am J Obstet Gynecol.

[21] Milne JL, Moore KN (2006). Factors impacting self-care for urinary incontinence. Urol Nurs.

[22] Xu D, Wang X, Li J, Wang K (2015). The mediating effect of 'bothersome' urinary incontinence on help-seeking intentions among community-dwelling women. J Adv Nurs.

[23] Björk AB, Sjöström M, Johansson EE, Samuelsson E, Umefjord G (2014). Women's experiences of internet-based or postal treatment for stress urinary incontinence. Qual Health Res.

[24] Asklund I, Samuelsson E, Hamberg K, Umefjord G, Sjöström M (2019). User experience of an app-based treatment for stress urinary incontinence: qualitative interview study. J Med Internet Res.

[25] Richter HE, Burgio KL, Chai TC, Kraus SR, Xu Y, Nyberg L (2009). Predictors of outcomes in the treatment of urge urinary incontinence in women. Int Urogynecol J Pelvic Floor Dysfunct.

[26] Labrie J, Lagro-Janssen AL, Fischer K, Berghmans LC, van der Vaart CH (2015). Predicting who will undergo surgery after physiotherapy for female stress urinary incontinence. Int Urogynecol J.

[27] Obloza A, Teo R, Marriott E, Parker G, Tincello D (2019). Association of baseline severity of lower urinary tract symptoms with the success conservative therapy for urinary incontinence in women. Int Urogynecol J.

[28] Carroll JK, Moorhead A, Bond R, LeBlanc WG, Petrella RJ, Fiscella K (2017). Who uses mobile phone health apps and does use matter? A secondary data analytics approach. J Med Internet Res.

